# Experience with pegvisomant treatment in acromegaly in a single Brazilian tertiary reference center: efficacy, safety and predictors of response

**DOI:** 10.1590/2359-3997000000210

**Published:** 2016-09-26

**Authors:** Leandro Kasuki, Evelyn de Oliveira Machado, Liana Lumi Ogino, Maria Caroline Alves Coelho, Cintia Marques dos Santos Silva, Luiz Eduardo Armondi Wildemberg, Carlos Henrique Azeredo Lima, Mônica R. Gadelha

**Affiliations:** 1 Faculdade de Medicina Hospital Universitário Clementino Fraga Filho Universidade Federal do Rio de Janeiro Rio de Janeiro RJ Brasil Faculdade de Medicina, Centro de Pesquisa em Neuroendocrinologia, Serviço de Endocrinologia, Hospital Universitário Clementino Fraga Filho, Universidade Federal do Rio de Janeiro (HUCFF/UFRJ), Rio de Janeiro, RJ, Brasil; 2 Laboratório de Genética Molecular Instituto Estadual do Cérebro Paulo Niemeyer Rio de Janeiro RJ Brasil Serviço de Neuroendocrinologia, Laboratório de Genética Molecular, Instituto Estadual do Cérebro Paulo Niemeyer, Rio de Janeiro, RJ, Brasil; 3 Hospital Federal de Bonsucesso Rio de Janeiro RJ Brasil Serviço de Endocrinologia, Hospital Federal de Bonsucesso, Rio de Janeiro, RJ, Brasil; 4 Hospital Pedro Ernesto Rio de Janeiro RJ Brasil Serviço de Endocrinologia, Hospital Pedro Ernesto, Rio de Janeiro, RJ, Brasil; 5 Instituto Estadual de Diabetes e Endocrinologia Luiz Capriglione Rio de Janeiro RJ Brasil Serviço de Endocrinologia, Instituto Estadual de Diabetes e Endocrinologia Luiz Capriglione (IEDE), Rio de Janeiro, RJ, Brasil

**Keywords:** Acromegaly, pegvisomant, growth hormone isoform

## Abstract

**Objective:**

To describe the safety and efficacy of pegvisomant therapy and the predictors of treatment response in acromegaly patients at a single tertiary reference center in Brazil.

**Materials and methods:**

We retrospectively reviewed the clinical, hormonal and radiological data of acromegaly patients treated with pegvisomant in our center. We also evaluated the presence of the d3 isoform of the growth hormone receptor (d3GHR).

**Results:**

Twenty-seven patients were included (17 women). Pegvisomant was used in combination with octreotide LAR in 20 patients (74%), in combination with cabergoline in one (4%) and as monotherapy in six (22%). IGF-I normalization was achieved in 23 patients (85%). Mild and transitory elevation of liver enzymes was observed in two patients (7.4%), tumor growth in one (3.4%) and lipodystrophy in two (7.4%). One patient stopped the drug due to headaches. The GHR isoforms were evaluated in 14 patients, and the presence of at least one d3GHR allele was observed in 43% of them, but it was not a predictor of treatment response. Only pre-treatment IGF-I level was a predictor of treatment response.

**Conclusion:**

Pegvisomant treatment was highly effective and safe in our series of Brazilian patients. A better chance of disease control can be expected in those with lower pre-pegvisomant IGF-I levels.

## INTRODUCTION

Acromegaly is a rare disease resulting from hypersecretion of growth hormone (GH) and as a consequence of insulin like growth factor-I (IGF-I), which in most cases is caused by a GH-secreting pituitary adenoma (1). Uncontrolled acromegaly is associated with increased morbidity and mortality (2,3). Surgery is the primary treatment in most cases, but approximately half of the patients will not be cured by the surgical procedure and therefore will need adjuvant medical therapy (4,5).

Three drug classes are currently available for acromegaly therapy: somatostatin analogues (SA), dopamine agonists (DA) and GH receptor (GHR) antagonists (4,6). Somatostatin analogues are considered the first option of medical treatment in the majority of patients, but prospective randomized studies show control rates of 20-40% for patients with first-generation SA (4,7-11). Pasireotide LAR, a next-generation SA (not yet approved for acromegaly treatment in Brazil), allows disease control in a higher percentage of patients and is effective in approximately 15% of those patients not controlled by first-generation SA with the dose of 40 mg and in 20% of the patients with the dose of 60 mg (12,13). The efficacy of cabergoline as monotherapy has not been evaluated in prospective studies, but normalization of IGF-I was reported in 34% of the patients in a meta-analysis of the literature, but can be as low as 10% in more recent studies, therefore, it is reserved for those patients with mildly elevated GH and IGF-I levels (4,14-16). The efficacy of other DA, like bromocriptine, is probably lower than that of cabergoline (4). These two drug classes (SA and DA) act on somatotropinoma through binding to its receptors (17,18).

Pegvisomant is the only drug in the GHR antagonist class and acts by binding to the GHR without triggering its intracellular pathways (19). It can be used in monotherapy or in combination therapy with SA, in this case with greater efficacy. Normalization of IGF-I levels was achieved in more than 90% of the patients in the initial clinical trials and in 63.2% of the patients in the last update report of the Acrostudy (20-22). Therefore, it is the most effective drug in controlling IGF-I levels in acromegaly.

There are no robust predictors of the response to pegvisomant treatment, although pre-treatment GH and IGF-I levels, gender, body mass index, fat mass, presence of type 2 diabetes mellitus (DM), age and previous radiotherapy can influence the chance of disease control (23,24). Additionally, some studies evaluated the GHR polymorphisms as a possible cause of a lower response to the drug, with conflicting results regarding whether patients who present the exon-3 deleted GHR (d3GHR) had a better response to pegvisomant (25-28).

Although an important tool for the management of acromegaly, pegvisomant treatment has never been described in a Brazilian multiethnic population, likely due to the limited availability of the drug in Brazil, considering that the treatment is not reimbursed by the Brazilian government. Therefore, the aim of this study was to describe the safety and efficacy of pegvisomant therapy and predictors of the treatment response in acromegaly patients at a single tertiary reference center in Brazil.

## MATERIALS AND METHODS

### Study population

We retrospectively reviewed the databank of acromegaly patients treated at the endocrinology outpatient clinic of the Hospital Universitário Clementino Fraga Filho (HUCFF), Universidade Federal do Rio de Janeiro (UFRJ) and selected those who were treated with pegvisomant for at least three months.

The diagnosis of acromegaly was made according to clinical and laboratory features, including increased serum IGF-I levels, according to the age and lack of GH suppression to less than 1.0 µg/L during the 75 g oral glucose tolerance test (4). Sellar magnetic resonance imaging revealed a pituitary adenoma in all of the patients.

#### Clinical, hormonal, radiological and treatment data

We collected the following data: age, sex, GH and IGF-I levels and tumor size at diagnosis and at the beginning of pegvisomant treatment, previous surgery and/or radiotherapy, previous acromegaly treatment, concomitant acromegaly treatment, duration of pegvisomant treatment and maximal pegvisomant dose.

#### Safety data

We reviewed data of the MRI and liver enzymes before and during pegvisomant treatment. We also reviewed the possible other side effects related to the drug such as lipodystrophy.

## Treatment protocol

For all patients who are treated with pegvisomant in the endocrinology outpatient clinic of HUCFF/UFRJ, a pituitary MRI is obtained immediately before the beginning of the treatment, and GH and IGF-I levels, liver enzymes (alanine aminotransferase and aspartate aminotransferase) and glucose levels are measured. Pegvisomant is started at a dose of 10 mg/day, and the dose is increased by 5 mg in consecutive increments every month of treatment until normal age-matched IGF-I levels are obtained (4). Somatostatin analogues are maintained if there is a biochemical (at least 20% of GH and/or IGF-I reduction) and/or tumor response (tumor stabilization or reduction > 20%) with the drug. In the absence of a response to SA, pegvisomant is started as a monotherapy. The dosage of the previous SA treatment is not changed when combination therapy with pegvisomant is initiated. In those patients with concomitant SA treatment and good control with low dose pegvisomant (10 mg/day), a weekly dose of 60 mg is implemented with a subsequent reduction to the minimal weekly dose, which is sufficient to maintain the IGF-I levels in the mid-range of the normal reference for appropriate age. For safety reasons, liver enzymes are measured monthly for the first six months and then each three months for six months and biannually thereafter. The pituitary MRI is repeated after six months of treatment and then annually. Efficacy was evaluated considering the last visit IGF-I level.

## Hormone assays

Plasma GH levels were measured by a chemiluminescence assay kit (IMMULITE 2000; DPC – Diagnostic Products Corp., Inc., Los Angeles, CA, USA). The inter- and intra-assay coefficients of variation (CV) were 6.0 and 5.8%, respectively. The International Reference Preparation (IRP) for GH was 98/574. The plasma IGF-I levels were measured by a chemiluminescence assay kit (IMMULITE 2000; DPC). The inter- and intra-assay CV were 6.6 and 3.6%, respectively (29). The IRP for IGF-I was 87/518. The IGF-I level was expressed as a percentage of the ULNR.

## Growth hormone receptor genotyping

The DNA was extracted from blood leukocytes with the Gentra Puregene Blood Kit (Qiagen, Valencia, CA, USA) according to the manufacturer’s protocol, and genotyping of GHR polymorphisms was conducted as follows:

A polymerase chain reaction (PCR) was performed with the following primers: one sense (G1: 5’-TGTGCTGGTCTGTTGGTCTG-3’) and two antisenses (G2: 5’-AGTCGTTCCTGGGACAGAGA-3’ and G3: 5’-CCTGGATTAACACTTTGCAGACTC-3’) [GenBank: AF 155912]. Briefly, the PCR was conducted in a 25 μL reaction mix using Hotstar Taq DNA polymerase (Qiagen) with denaturation at 94 °C for 5 min, followed by 35 cycles of 94 °C for 30 sec, 60 °C for 30 sec, and 72 °C for 90 sec, and a final extension phase at 72 °C for 7 min. Then, the reaction products were run with ethidium bromide-stained 2% agarose gel electrophoresis. A full-length GHR allele (flGHR) was shown by the presence of two bands of approximately 935 bp. In the presence of the genomic deletion of exon 3 (d3GHR), a 532-bp band was formed.

## Statistical analysis

The statistical analyses were performed using SPSS version 20.0 for MacOS (SPSS Inc., Chicago, IL). For the descriptive analysis, categorical variables were expressed as the percentage and frequency, and the numerical variables were expressed as the mean ± DP or median (min – max) according to the distribution of the sample. The difference between the IGF-I levels before and after pegvisomant therapy was evaluated by the Wilcoxon test. The Spearman test was used for correlations. A p*-*value < 0.05 was considered statistically significant.

## RESULTS

### Characterization of the study population

#### Clinical, biochemical and previous treatment characteristics

A total of 27 patients [17 women (63%)] were enrolled in the study. The mean age at diagnosis was 41.3 ± 16.7 years. Twenty-one patients (78%) were submitted to surgery and 11 to radiotherapy (41%). A macroadenoma was observed during diagnosis in 25 patients (93%). Median GH and IGF-I levels during diagnosis were 14.2 µg/L (3.4 – 252.0) and 295% ULNR (157 – 671), respectively. Twelve patients (44%) presented diabetes mellitus before pegvisomant treatment.

Octreotide LAR treatment was attempted before pegvisomant in 26 patients (96%). It was the primary therapy in 6 patients: two patients presented high surgical risk and four patients presented tumors that were mainly located in the cavernous sinus. The maximal dose was 30 mg every 28 days in 16 patients and 40 mg every 28 days in 10 patients. The association of cabergoline to octreotide LAR treatment was also attempted in 21 of the 27 patients (78%).

## Pegvisomant treatment

### Efficacy

The median GH and IGF-I levels before pegvisomant treatment were 3.7 µg/L (0.8 – 209.0) and 212% ULNR (132 – 637), respectively. Pegvisomant was used in combination with octreotide LAR in 20 patients (74%) and in combination with cabergoline in one patient (4%). The dose of octreotide LAR was 20 mg in one patient and 30 mg in the remaining 19 patients during combination therapy. The dose of cabergoline was 3.0 mg/week in the only patient who used cabergoline in association with pegvisomant. In six patients, pegvisomant was used as single treatment. The median treatment duration was 15 months (3 – 69 months), and the median pegvisomant dose was 10 mg/day [6 (40 mg/week) – 30 mg/day].

Acromegaly control (normalization of IGF-I) was obtained in 23 patients (85%). The normalization of IGF-I was obtained in five out of six patients in monotherapy (83%) and in 18 out of 21 patients (86%) in combination therapy. In three patients, a transition to a weekly dose of PEG was possible (ranging from 40 – 70 mg/week) with maintenance of normal IGF-I levels. The median IGF-I levels after treatment were 76% ULNR (47 – 308). The IGF-I levels presented a median reduction of 66% (0 – 82) from pre-treatment values. The median dose of pegvisomant was 10 mg/day (6 – 20 mg/day) in the controlled patients, whereas it was 22.5 mg/day (10 – 30 mg/day) in the uncontrolled patients.

### Predictors of response

There was no difference in the age (at diagnosis or at the moment of pegvisomant initiation), sex, frequency of previous radiotherapy or GH or IGF-I levels at diagnosis between patients who were controlled and those who were not after pegvisomant treatment (Table 1).

**Table 1:** Comparison of clinical, biochemical and treatment characteristics between patients controlled or not during pegvisomant treatment

Variable	Controlled	Not controlled	p-value
Age at diagnosis (years)	36 (20 – 75)	41 (28 – 82)	0.576
Age at the moment of PEG initiation	47 (24 – 84)	49 (36 – 86)	0.705
Female sex (%)	39	25	1.000
Previous radiotherapy (%)	43	25	0.624
GH levels at diagnosis (μg/L)	10.6 (3.4 – 198.0)	35.2 (15.8 – 252.0)	0.145
IGF-I levels at diagnosis (%ULNR)	300 (157 – 671)	295 (182 – 322)	0.635
GH levels pre-PEG (μg/L)	2.8 (0.7 – 17.3)	6.5 (2.6 – 209.0)	0.095
IGF-I levels pre-PEG (%ULNR)	208 (132 – 390)	559 (214 – 637)	0.004

% ULNR: percentage of the upper limit of normal range.

The median GH level before pegvisomant treatment was 2.8 µg/L (0.7 – 17.3) in those patients controlled after treatment, whereas it was 6.5 µg/L (2.6 – 209.0) in non-controlled patients, although the difference did not reach statistical significance (p = 0.095). The pre-treatment median IGF-I levels were lower in those patients controlled after pegvisomant treatment than in those not controlled after treatment [208% ULNR (132 – 390) vs. 559% ULNR (214 – 637), p = 0.004] (Table 1).

There was no correlation between the age at diagnosis, pre-treatment GH or IGF-I levels with the percentage of IGF-I reduction after treatment.

Growth hormone receptor polymorphisms were studied in 14 patients. The flGHR was observed in both alleles in eight patients (57%). Two patients (14%) were homozygous and four patients (29%) was heterozygous for the d3GHR allele. There was no difference in age, GH or IGF-I levels before pegvisomant treatment or in the percentage of IGF-I reduction between homozygous flGHR patients and those who were not. Additionally, there was no difference in the frequency of IGF-I normalization between patients homozygous for the flGHR and those who were not.

### Safety

**Figure 1:**
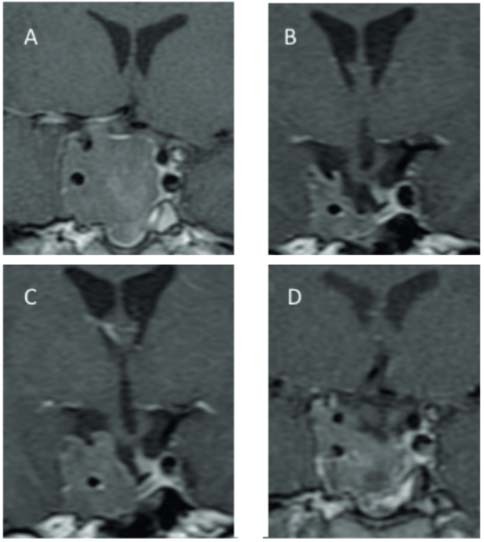
Sellar magnetic resonance imaging (T1 weighted after gadolinium enhancement) showing the growth of a residual tumor located mainly in the right cavernous sinus during pegvisomant treatment, that was sustained after drug withdrawal: (A) at diagnosis; (B) before pegvisomant treatment; (C) during pegvisomant treatment; (D) six months after pegvisomant withdrawal.

Tumor enlargement was observed in one patient (3.7%), but tumor growth continued despite withdrawal of pegvisomant (Figure 1). This was a young patient with an aggressive tumor since diagnosis (the tumor presented a Ki-67 labeling index of 4.0% and a p53 of 6.0%). The patient was submitted to surgery and treated with octreotide LAR 30 mg every 4 weeks before pegvisomant treatment. She was then treated with a combination therapy (octreotide LAR + pegvisomant), and after tumor enlargement, pegvisomant was withdrawn, but the tumor continued to grow. The patient was submitted to another surgery and radiotherapy with IGF-I normalization.

Elevated liver enzymes were observed in two patients (7.4%) but were mild (1.5 and 2.3x the ULNR), transient (two and five months) and reverted despite pegvisomant maintenance. Both patients were in combination treatment with pegvisomant and octreotide LAR. No patient presented liver failure or required drug withdrawal due to hepatic side effects.

Lipohypertrophy was observed in two patients (7.4%); both were non-controlled with pegvisomant therapy and reversed after reeducation regarding the importance of the rotation of the drug injection sites.

One patient presented a headache that was possibly related to the drug, as it ceased when the drug was stopped and recurred when pegvisomant was reintroduced, which led to the suspension of the treatment. This patient was being treated with pegvisomant as monotherapy and was the only patient in whom the drug was withdrawn due to a drug-related side effect.

No other drug-related side effects were observed.

## DISCUSSION

We demonstrated that pegvisomant treatment is effective and safe for the first time in a cohort of Brazilian acromegaly patients to accompany descriptions in other populations. Additionally, we explored possible predictors of the treatment response, and only pre-treatment IGF-I levels were predictive of disease control with pegvisomant.

Acromegaly is associated with enhanced mortality and a high morbidity when normalization of GH and IGF-I levels is not achieved (2). Although surgery and tumor-directed drugs (SA and DA) permit disease control in the majority of patients, there are some cases that require additional treatments (4,30). In our series, the majority of patients (78%) were submitted to surgery, and all but one patient was treated with first-generation SA, with association with cabergoline tried in 78%. No patient received pasireotide LAR. Radiotherapy was administered to 41% of the patients. However, despite all of these treatments, they maintained elevated GH and IGF-I levels.

Pegvisomant is highly effective in normalizing IGF-I levels, even in patients resistant to other treatments (30), and this was confirmed in our series with a high percentage (85%) of disease control. Our results are closer to the data reported in the initial clinical trials with pegvisomant monotherapy and those from series of other tertiary centers than to the data reported in the last update of the Acrostudy (20-22,31,32). One of the possible reasons for a lower control rate in the Acrostudy is the clinical inertia (22). As the databank accepts inclusions from many centers, there are probably some centers with less experience in treating acromegaly and therefore in adjusting treatment to attain the goals of disease control. Because we are a tertiary reference center, drug escalation and the pursuit to obtain disease control is probably more intensive (22). Another possible explanation is the higher number of patients treated with a combination of pegvisomant and SA (74%) than that observed in the Acrostudy (37%), as the reported control rates with combination therapy can be as high as 97% in other studies (33,34). It is also important to highlight that in the Acrostudy, IGF-I normalization was recorded on annual bases, therefore it can provide lower control rates than studies assessing normalization of IGF-I at the last patient visit or at any time during follow-up.

The safety profile of the drug in our patients was also similar to what is reported the literature (22) with only mild elevations of liver enzymes and lipohypertrophy. Only one patient presented tumor growth during treatment, but this patient harbored an aggressive tumor that continued to grow after the drug was withdrawn. There were no cases of serious adverse effects that required suspension of the drug.

There are few studies in the literature addressing predictors of response to pegvisomant treatment (23,24). One of the possible predictors is the presence of the d3GHR isoform of the GHR. Two initial studies, including 19 and 44 patients, described that in the presence of the d3GHR isoform, a lower dose of pegvisomant and a shorter treatment was necessary to obtain normalization of IGF-I levels. However, Filopanti and cols. (27) in a larger multicenter study (111 patients) did not find a difference in the response rates to pegvisomant treatment between those patients presenting d3GHR or the flGHR. Additionally, Franck and cols. (28) recently described no difference in the response rates during combination therapy with pegvisomant and SA in patients presenting the different isoforms of GHR. Our results are in accordance with these larger studies in the literature; we also found no difference in the treatment response rates.

The only predictor of response in our series was the pre-pegvisomant IGF-I level. Although pre-pegvisomant GH levels were lower in those patients controlled after pegvisomant treatment than in the non-controlled patients, the difference was not statistically significant (p = 0.095). As we mentioned, we also did not find a difference in the control rate in the presence of the d3GHR isoform. However, considering the number of patients in our series, a type II error cannot be excluded.

In conclusion, treatment with pegvisomant was highly effective and safe in our series of Brazilian patients as previously reported in other populations. A better chance of disease control can be expected in those with lower pre-pegvisomant IGF-I levels, and no difference was observed in the presence or absence of the d3GHR isoform.
